# Stigmatizing attitudes and misconceptions about obesity among Spanish healthcare professionals

**DOI:** 10.1371/journal.pone.0351868

**Published:** 2026-06-18

**Authors:** David Sánchez-Carracedo, Albert Fornieles-Deu, Dimitra Anastasiadou, Stuart W. Flint

**Affiliations:** 1 Department of Clinical and Health Psychology, Eating and Weight-Related Problems Unit, Universitat Autònoma de Barcelona, Barcelona, Spain; 2 Department of Psychobiology and Methodology of Health Sciences. Eating and Weight-Related Problems Unit. Universitat Autònoma de Barcelona, Barcelona, Spain; 3 Department of Clinical and Health Psychology. Eating and Weight-Related Problems Unit, Universitat Autònoma de Barcelona, Barcelona, Spain; 4 School of Psychology, University of Leeds, Leeds, United Kingdom; 5 Scaled Insights, Nexus, Leeds, United Kingdom; Ulster University - Coleraine Campus: Ulster University, UNITED KINGDOM OF GREAT BRITAIN AND NORTHERN IRELAND

## Abstract

Weight stigma and misconceptions about obesity among healthcare professionals (HCPs) may negatively affect healthcare quality and access for people living with obesity (PLWO). However, no previous research has examined these attitudes and beliefs in Spanish HCPs. This cross-sectional online study examined weight stigma and obesity-related beliefs among 922 HCPs recruited via Spanish professional and academic obesity-related organizations ((74.1% female, mean BMI = 23.73 kg/m^2^ (SD 3.89), mean age = 43.73 years (SD 12.45), range 23–75, 88.7% provide care for PLWO)). Anti-fat attitudes were measured using the Dislike subscale of the Anti-Fat Attitudes questionnaire (AFA) and the Fat Phobia Scale (F-Scale). ANOVAs adjusted for sociodemographic variables, weight status, and weight bias internalization were conducted. Overall, Spanish HCPs surveyed reported negative attitudes toward PLWO. Lower weight status was consistently associated with higher stigma scores. Younger age (F-Scale) and working in the private sector (Dislike) were associated with higher scores. Differences across specialties were observed, with obesity physicians reporting the lower stigma levels. A substantial proportion of HCPs endorsed beliefs emphasizing personal responsibility: 38% attributed overeating to individual causes, 66% believed obesity could be entirely prevented by a healthy lifestyle, and 59% believed it could be cured through lifestyle changes. Many also attributed weight loss difficulties, poor compliance, and weight regain to lack of motivation and lifestyle choices, and over half considered lifestyle or psychological interventions the most effective treatment for severe obesity. Endorsement of these beliefs was consistently associated with higher stigma scores. These findings provide national evidence that weight stigma among Spanish HCPs is present and linked to beliefs framing obesity as primarily under individual control. These attitudes and knowledge gaps may contribute to inappropriate care and unfair treatment of PLWO. Addressing responsibility-focused beliefs may be a key step towards reducing stigma and improving the quality of obesity care in Spain.

## Introduction

As obesity rates‌‌ have increased worldwide [1], so too has weight biases against individuals with higher body weight [2]. Among adults with overweight or obesity (based on BMI categories), the prevalence of weight stigma ranges from 19–42%, with higher rates observed among those with a higher body mass index (BMI) and among women compared to men [3–5]. A multinational study conducted in Australia, Canada, France, Germany, United Kingdom (UK), and United States (US) revealed that 56–61% of adults with obesity participating in weight loss programs reported experiencing weight-related stigma, with two-thirds in each country reporting experiencing weight stigma from doctors [6].

Weight stigma is now widely recognized as both a global health challenge and a human rights issue [7,8] and is associated with adverse psychological outcomes, disordered eating, reduced engagement in health behaviors, and poorer weight-related outcomes [9–15]. Furthermore, when weight stigma is internalized, which involves applying negative weight-related stereotypes to oneself, leading to self-devaluation [16], it can be associated with poorer mental health outcomes, disordered eating, weight gain, and decreased motivation and self-efficacy for engaging in health-promoting behaviors [9].

Weight-based stigma is particularly concerning within healthcare settings. When healthcare professionals (HCPs) are the source, stigma may affect consultations, diagnostic procedures, and patient interactions [3,17], and may discourage individuals from seeking care due to fear of prejudice and internalized weight bias [18,19], contributing to delayed diagnoses and poorer outcomes [20,21].

A recurring finding is that many HCPs attribute obesity primarily to behavioral causes such as lack of willpower or personal responsibility, overlooking the multifactorial interplay of biological, genetic, environmental, and socio-economic factors [22,23]. Such attributions may contribute to less patient-centered communication and lower quality care practices [20,24], reflect a weight-normative approach observed among HCPs and students [24–26], and paradoxically undermine patients’ motivation and engagement in health behaviors [27]. In addition, many professionals report feeling unprepared to manage obesity and perceive treatment as ineffective [19,28].

International research across diverse health professions — including physicians, nurses, dietitians, psychologists, and allied health professionals [29] — consistently documents both implicit and explicit weight bias in healthcare settings [24,30–35] as well as similar patterns among undergraduate students in health-related majors [25,26]. Evidence also suggests that higher stigma levels are associated with male gender, younger age, and lower BMI among HCPs, although findings are not entirely consistent [24,30,36–39]. However, despite this growing international literature, evidence specific to Spain remains scarce.

The present study builds in part on the framework proposed by the ASK Study (Attitudes, Stigma and Knowledge) [24], one of the largest multinational investigations examining HCPs’ attitudes and beliefs about obesity among HCPs from 77 countries worldwide, employing the Fat Phobia Scale (F-Scale) and a tailored questionnaire to explore beliefs about the causes, prevention, and treatment of obesity, attitudes toward available treatments, and research priorities. Notably, approximately 60% of HCPs believed that obesity could be prevented and treated through a healthy lifestyle, while these beliefs were associated with higher weight stigma.

To our knowledge, only two studies examining HCPs’ perceptions and attitudes toward obesity have included Spanish HCPs: the ACTION-IO study [40] and the ACTION Teens Global survey study [41] Both of these studies examined HCPs’ perceptions, attitudes, behaviors, and perceived barriers to effective obesity care of PLWO. Caterson et al.[40] recruited 2,785 HCPs from 11 countries (306 from Spain) and Halford et al.[41] recruited 2,323 HCPs from 10 countries (251 from Spain). Notably, the ACTION-IO study revealed that despite recognizing obesity as a disease impacting overall health, one-third of HCPs placed the responsibility for weight loss on individuals with obesity. Regarding the previously cited ASK Study [24] conducted with HCPs from 77 countries worldwide, data were not provided about the 77 countries HCPs resided in. None of these studies, all of which are multinational, differentiate results by country. Therefore, it remains unclear whether the patterns observed internationally — particularly the association between responsibility-focused beliefs and weight stigma — apply within the Spanish healthcare context. The current study aimed to examine HCPs perspectives on obesity for the first time in Spain, with tow specific objectives: [1] to assess attitudes towards obesity in Spanish HCPs and their association with sociodemographic variables; and [2] to examine whether the beliefs of Spanish HCPs regarding the causes and treatments of obesity and their association with their attitudes.

## Materials and methods

### Design and Participants

This study used a cross-sectional online survey-based design and is reported in accordance with the STROBE (Strengthening the Reporting of Observational Studies in Epidemiology) guidelines for cross-sectional studies (see S1 Checklist). This study relied on a non-probabilistic convenience sample of HCPs recruited through professional scientific societies. Participation was voluntary. As the survey link was distributed by the participating societies to their members, the research team did not have access to the total number of individuals who received the invitation; therefore, a response rate could not be calculated. Eligibility criteria were minimal. Participants were required to be affiliated with one of the participating scientific societies and to provide informed consent before completing the survey. No additional inclusion or exclusion criteria were imposed. A total of 922 HCPs from different specialties participated. Of these, 683 (74.1%) were women. Mean BMI was 23.73 (SD = 3.89), and mean age was 43.73 years (SD = 12.45, range 23–75). The most frequent specialty was pediatrics (36.4%), and 89.1% provided care for PLWO. Table 1 shows the main sociodemographic characteristics of the sample.

**Table 1:** Description of the sociodemographic variables of the sample, weight status, and Dislike, F-Scale and WBISM scores for each level of the sociodemographic and weight status variables.

Variables	Category	N (%)	Dislike	F-Scale
			**M (SD)**	**M (SD)**
Profession/ Speciality	General practitioner	94 (10.2)	2.10 (0.91)	3.58 (0.46)
	Obesity physician	31 (3.4)	1.81 (1.04)	3.44 (0.57)
	Bariatric surgeon	41 (4.4)	2.21 (0.93)	3.64 (0.33)
	Endocrinologist/ Diabetologist	141 (15.3)	2.15 (0.86)	3.55 (0.44)
	Internal Medicine	109 (11.8)	1.97 (0.88)	3.45 (0.52)
	Paediatrician	335 (36.3)	2.17 (0.92)	3.56 (0.43)
	Other	171 (18.5)	2.02 (0.81)	3.46 (0.39)
Workplace*	Private sector	140 (15.3)	1.91 (0.92)	3.47 (0.48)
	Public sector	776 (84.7)	2.02 (0.81)	3.54 (0.43)
Providing care for PLWO	Yes	818 (88.7)	2.10 (0.88)	3.53 (0.45)
	No	104 (11.3)	2.11 (0.97)	3.49 (0.37)
Gender	Women	683 (74.1)	2.09 (0.88)	3.53 (0.44)
	Men	239 (25.9)	2.12 (0.93)	3.52 (0.45)
Weight status**	Underweight (≤ 18.5 kg/m^2^)	30 (3.3)	2.10 (0.77)	3.64 (0.37)
	Normal range (18.5–24.9 kg/m^2^)	620 (67.2)	2.17 (0.93)	3.55 (0.45)
	Overweight (25.0–29.9 kg/m^2^)	208 (22.6)	1.98 (0.79)	3.49 (0.41)
	Obesity (≥ 30.0 kg/m^2^)	61 (6.6)	1.81 (0.79)	3.36 (0.43)
Age	< 35 years	270 (29.3)	2.12 (0.85)	3.60 (0.44)
	35-44 years	248 (26.9)	2.06 (0.91)	3.53 (0.49)
	45-54 years	183 (19.8)	2.01 (0.87)	3.51 (0.41)
	≥ 55 years	221 (24.0)	2.19 (0.93)	3.45 (0.39)
Dislike			2.10 (0.89)	
F- Scale				3.53 (0.44)
WBISM			2.12 (1.08)	

Note: M: Mean; SD: Standard Deviation; CI 95%: Confidence Interval 95%; PLWO: people living with obesity. F-Scale: Fat Phobia Scale. WBISM: Modified Weight Bias Internalization Scale. *Additionally, six participants (0.65%) selected “Other” as workplace. ** The underweight and normal range categories have been unified for further analysis due to the low representation of the underweight category (0.32%).

### Procedure

The first author requested the ASK inventory (attitudes, stigma, and knowledge on obesity and type 2 diabetes) from Dr. Stuart W. Flint, an author of the ASK study [24], to replicate the study in Spain. An adaptation of the selected questions for this study was made in Spanish, specifically designed by the ASK study authors to investigate beliefs about the causes and available treatments for obesity, as well as attitudes toward obesity. At the beginning of July 2020, the first author of the current study contacted the main scientific societies of registered HCPs in the Spanish Ministry of Health, Consumer Affairs, and Social Welfare in the following areas: Endocrinology and Nutrition; Primary Care/ Family and Community Medicine; Internal Medicine; Preventive Medicine and Public Health; and Pediatrics. Additionally, other Spanish scientific societies, not included in this register but linked to the study of obesity, were also contacted. These scientific societies shared a link to the survey with all their members, endorsing the study’s interest. Individuals who were interested in participated followed the link to the survey, where they were presented with the study information sheet and informed consent. Individuals who agreed to participate, provided written online consent. Responses were collected until October 21, 2020 (see acknowledgements section for the list of associations that agreed to participate in the study and sent the survey link to their members). The survey was hosted in Google Forms where potential participants were directed if they selected the link. They were asked to read an information sheet and provide informed consent before starting the survey if they wished to take part. As in the ASK study, a forced-response design was used for all survey questions except weight and height and questions seven and eight from the inventory adapted from the ASK study. Only three participants did not report their weight. This study was conducted in accordance with the guidelines established in the Declaration of Helsinki of the World Medical Assembly [42] and was approved by the Ethics Committee of the first author´s university (CEAAH 3451).

### Measures

#### Sociodemographic and anthropometrics.

Participants reported information about gender, age, workplace, providing care for PLWO, weight and height. The profession (health specialty) was collected in the following same 12 categories as in the ASK study: general practitioner, obesity physician, bariatric surgeon, endocrinologist/diabetologist, internal medicine, psychologist/psychiatrist, registered dietician, other allied health professional, commissioner/payer, public health specialist, healthcare manager/administrator, and other (open question). Due to the low representation of some categories, they have finally been grouped into the following seven: general practitioner, obesity physician, bariatric surgeon, endocrinologist/diabetologist, internal medicine, pediatrician, other.

Weigh status was calculated using the World Health Organization (2007) BMI cut-offs standards [43] (underweight <18.5 kg/m^2^, normal range 18.5–24.99 kg/m^2^, overweight 25–29.99 kg/m^2^, obesity ≥ 30 kg/m^2^). We use this criterion to facilitate international comparisons, but we want to highlight the major limitations associated with the use of BMI as a health indicator [44].

#### Weight bias internalization (WBI).

WBI has been defined as the process of being aware and agreeing with negative weight-based stereotypes, applying these to oneself and engaging in self-blame and self-devaluation for weight [16]. WBI is referred to a self-stigma, while anti-fat attitudes refer to public stigma, and there are conceptual differences between both types of stigmas. The social depreciation is related to the public stigma, directed at others, such as public stereotyping attitudes, emotional reactions about such stereotypes, and discriminatory behaviors due to prejudice, that characterize anti-fat attitudes (interpersonal component), while WBI is related to self-stigma (intrapersonal component) [45]. Despite the conceptual differences among them, as WBI has been correlated with anti-fat attitudes [46–48], we decided to assess WBI as possible confounder in our study. We used the Modified Weight Bias Internalization Scale (WBISM) [47] in its Spanish validation for adults [49]. The WBISM measures weight self-stigma across the body weight statuses (e.g., “I hate myself for my weight”). The Spanish version used has 11 items with responses rated on a 7-point Likert scale, ranging from 1 (strongly disagree) to 7 (strongly agree). The mean of the item responses serves as the participant’s score (range 1–7), with higher scores indicating higher internalized weight bias. The Spanish validation for adults of WBISM has shown a high internal consistency (Cronbach’s alpha ranged from.927 to.935 and McDonald’s omega ranged from.929 to.937), adequate t-test reliability (r = .879) and showed a unidimensional structure with an adequate fit. In our sample, WBISM has shown a high internal consistency (α = .88; ω = .91). According to previous research, WBISM scores were categorized above and below 4 for analytical purposes [50].

#### Anti-fat Attitudes.

The *Dislike subscale of the Anti-fat Attitudes Questionnaire* (AAQ-D; [51] in its Spanish version [52] was administered. It has 7 items (e.g., “I really don’t like fat people much”) with responses ranging from 1 (‘strongly disagree’) to 7 (‘strongly agree’). The mean of the item responses serves as the participant’s score (range 1–7), with higher scores indicating higher negative attitudes towards obesity. Cronbach’s alpha was among 0.70 and 0.86. In our sample, Cronbach’s alpha in our sample was.82 and ω = .79.

The *Fat Phobia Scale – Short form (F-Scale)* [53] assesses the extent to which participants associate stereotypical characteristics, such as being ‘lazy’ or ‘inactive,’ with obesity. We included the adapted Mexican Spanish version [54] of the F-Scale in addition to the validated Spanish version of the AFA Dislike subscale, to allow comparability with the ASK study and to assess weight stigma across complementary components using two conceptually related but distinct instruments. It consists of 14 opposing adjectives (e.g., ‘no willpower’/ ‘has willpower’) with scores ranging from 1 to 5. Higher scores are associated with adjectives with negative connotations, while lower scores correspond to positive connotations. The mean of the item responses serves as the participant’s score (range 1–5). We adapted the Mexican version [54] to Spanish in Spain. Minor wording adaptations were introduced to items 6, 11, and 12 to ensure linguistic appropriateness for the Spanish context, based on expert consensus within the research team. No formal psychometric validation of the adapted version was conducted in Spain. Internal consistency in the present sample was α = ..85; ω = .84.

#### Beliefs about causes and treatments of obesity.

These beliefs were evaluated through the following eight questions selected from the ASK study [24]: (Q1) “Obesity is best defined as…” (five response options); (Q2) “What is the most common cause of a person overeating?” (six response options); (Q3) “Which of the following conditions/diseases could be entirely prevented by an individual’s commitment to follow a healthy lifestyle?” (Yes/No response for six chronic diseases including obesity); (Q4) “Which of the following conditions/diseases could be cured by an individual’s commitment to follow a healthy lifestyle?” (Yes/No response for six chronic diseases including obesity); (Q5) “What makes it difficult for people to lose weight? (four response options); (Q6) “Which of the following is the most effective treatment for severe obesity (BMI >35 kg/m^2^)?” (four response options); (Q7) “A patient does not lose significant weight while participating in a lifestyle intervention programme. This is most likely due to...” (four response options); (Q8) “After losing a significant amount of weight from a lifestyle programme a patient regains all/most of their weight. This is most likely due to...” (four response options). The underlines are original. The original ASK inventory included twenty-six questions written by collaborative consensus by the research team and was not designed as a psychometrically validated scale, but to investigate beliefs about obesity, attitudes towards obesity and type 2 diabetes and knowledge of related therapies [24]. For the present study, we selected those eight questions whose answers included the belief that weight is easily controllable, and obesity derives from a lack of self-discipline and personal responsibility, one of the key factors associated with weight stigma together with negative societal perceptions of obesity [55]. The selected items were translated and linguistically adapted into Spanish by the research team to ensure contextual appropriateness.

### Data analysis

Statistical analyses were performed with IBM-SPSS 29 software. Sociodemographic and anthropometric characteristics, Dislike and F-Scale scores were described using number (n) and percentage (%) or mean and standard deviation (SD) as appropriate. For the first objective, ANOVAs were performed between the Dislike and F-Scale and sociodemographic variables (profession/specialty, workplace, providing care for PLWO, gender, and age) and weight status, using the remaining variables and WBISM as adjustment variables. For the second objective, ANOVAs were conducted between the Dislike and F-Scale, and selected questions from the ASK study, with sociodemographic variables, weight status, and WBISM as adjustment variables. All adjustment variables were categorical or categorized and were entered as fixed factors in the ANOVA (GLM) models. Homogeneity of variances was examined using Levene’s test. The significance level was set at.05. Effect size was measured using Cohen’s d for the comparison of two means and η² effect size for more than two means. They can be interpreted by means of Cohen’s criteria [56]; an Eta-Squared of 0.0099 relates to a Cohen “small effect (0.2); an η² of 0.0588 relates to a Cohen “medium effect (0.5); an Eta-Squared of 0.1379 relates to a Cohen “large effect” (0.8). For sociodemographic variables, *post hoc* comparisons were employed using Bonferroni adjustment when appropriate.

## Results

Table 1 shows the demographic and anthropometric characteristics of the sample. Regarding the profession/speciality, since the category ‘other’ was an open question and some responses had a high frequency, the first step was to review and group responses into seven new categories: pediatrician, pediatrician specialist, surgeon, pharmacist, nurse, researcher, others. Given the low frequency of some healthcare specialties, they were finally reduced to six. The original categories general practitioner, obesity physician, bariatric surgeon and endocrinologist/diabetologist were maintained. Pediatrician and pediatrician specialist were grouped into “pediatrician”. A final “other” category was created by grouping internal medicine, psychologist/psychiatrist, registered dietician, other allied health professional, commissioner/payer, public health specialist, healthcare manager/administrator, other, surgeon, pharmacist, nurse, and researcher. Slightly more than one-third of the sample were pediatricians, the most common specialty. Only three cases reported belonging to workplaces other than the public or private sector, so they were not included in the analyses adjusted for this variable. Almost 85% of participants worked in the public sector, and almost 90% provided care for PLWO. Almost 30% of participants were living with overweight or obesity. In general, HCPs exhibited slightly negative attitudes towards obesity, with F-Scale scores above 3 (M = 3.53, SD = 0.44; range 1.86–5). Dislike scores were M = 2.09 (SD = 0.88).

Table 2 shows the ANOVA of the relationship between weight stigma scales and sociodemographic variables. Regarding the F-Scale, significant differences were found based on Profession/Specialty, although the *post hoc* contrasts were not significant. Bariatric surgeons had the highest scores, while obesity physicians had the lowest. There were also significant differences based on weight status and age. HCPs with underweight-normal range had significantly higher scores than those with obesity (*p* = .002), and those aged ≤ 35 years had significantly higher scores than those aged ≥ 55 years (*p* = .001). Regarding the Dislike scale, there were also differences based on weight status, with higher scores in HCPs with normal-underweight compared to those with overweight (*p* = . 023) and those with obesity (*p* = .01). Additionally, those working in the public sector had significantly higher scores than those in the private sector. Although men showed slightly higher scores on Dislike, this difference did not reach statistical significance (*p* > .05).

**Table 2:** ANOVA of the relationship between Dislike and F-Scale scales, and sociodemographic variables.

Variable	Dislike			F-Scale		
F	*p*	Cohen’s d/ η²*	F	*p*	Cohen’s d/ η²*
Profession/Specialty	1.83	.091	.012	2.93	**.008**	.019
Workplace	7.26	**.007**	.008	1.96	.162	.002
Providing care PLWO	0.04	.840	.000	0.06	.808	.000
Weight status	15.56	**<.001**	.033	6.63	**.001**	.014
Gender	3.71	.054	.004	2.64	.105	.003
Age	2.29	.077	.008	3.42	.**017**	.011

Note: PLWO: people living with obesity. Adjusted by sociodemographic variables, weight status and WBIS-M. F = F statistic from the ANOVA models. Bold values indicate statistically significant results (*p* < .05). *d Cohen for the comparison of two means; η² effect size for more than two means. Post hoc comparisons were employed using Bonferroni adjustment when appropriate.

Table 3 shows the description of the main beliefs of HCPs regarding the causes and management of obesity, as well as their association with weight stigma scales. The most accepted definition of obesity by HCPs (Q1) is as ‘a risk factor for other diseases and sometimes a disease in itself’ (71.9%; n = 663). There were no differences in weight stigma levels based on how HCPs define obesity.

**Table 3:** Beliefs about causes and treatments of obesity and ANOVAs of their association with Dislike and F-Scale.

		Dislike				F-Scale			
	N (%)	M (SD)	*F*	*p*	d Cohen/ η²*	M (SD)	*F*	*p*	Cohen’s d/ η²*
**Q1. Obesity is** best **defined as…**									
			2.06	.084	.009		1.27	.282	.006
1. A lifestyle choice	7 (0.8)	3.00 (1.98)				3.17 (0.73)			
2. A disease	227 (24.6)	2.04 (0.85)				3.54 (0.43)			
3. A risk factor for other diseases and sometimes a disease in itself	663 (71.9)	2.10 (0.87)				3.53 (0.44)			
4. A risk factor for other diseases	14 (1.5)	2.12 (0.83)				3.58 (0.40)			
5. Other	11 (1.2)	1.99 (0.83)				3.47 (0.46)			
**Q2. What is the** most common **cause of a person overeating?**									
			2.34	**.040**	.013		2.91	**.013**	.016
1. Food environment	157 (17.0)	2.08 (0.74)				3.49 (0.43)			
2. Food addiction	36 (3.9)	2.38 (1.30)				3.69 (0.58)			
3. Gluttony	14 (1.5)	2.22 (0.58)				3.45 (0.66)			
4. Emotional/comfort eating	301 (32.6)	2.17 (0.96)				3.58 (0.43)			
5. Malfunction of physiological mechanisms that regulate appetite/satiety	333 (36.1)	1.98 (0.76)				3.48 (0.43)			
6. Other	81 (8.8)	2.14 (1.03)				3.54 (0.42)			
**Q2 (bis). What is the** most common **cause of a person overeating?**									
−			8.17	**.004**	.009		10.71	**.001**	.012
− Personal causes [2–4]	349 (38.1)	2.19 (0.99)				3.59 (0.46)			
− Other causes [1,5,6]	567 (69.1)	2.03 (0.80)				3.49 (0.53)			
**Q3. Which of the following conditions/diseases could be** entirely prevented **by an individual’s commitment to follow a healthy lifestyle?**									
− Type 1 diabetes			0.002	.968	.000		7.36	**.007**	.008
No	891 (96.6)	2.09 (0.88)				3.54 (0.43)			
Yes	31 (3.4)	2.08 (0.95)				3.30 (0.61)			
− Cancer			1.83	.177	.002		3.71	**.054**	.004
No	734 (79.6)	2.08 (0.86)				3.52 (0.43)			
Yes	188 (20.4)	2.15 (0.97)				3.56 (0.50)			
− Obesity			7.97	**.005**	.009		9.55	**.002**	.010
No	317 (34.4)	1.98 (0.80)				3.47 (0.41)			
Yes	605 (65.6)	2.15 (0.91)				3.56 (0.45)			
− HIV Infection			0.01	.915	.000		2.03	.151	.002
No	572 (62.0)	2.10 (0.90)				3.52 (0.45)			
Yes	350 (38.0)	2.09 (0.85)				3.55 (0.43)			
− Type 2 diabetes			2.18	.140	.002		3.96	.**048**	.004
No	354 (38.4)	2.04 (0.87)				3.50 (0.42)			
Yes	568 (61.6)	2.13 (0.89)				3.55 (0.46)			
− Osteoarthritis			0.10	.753	.000		0.73	.394	.001
No	738 (80.0)	2.09 (0.85)				3.54 (0.42)			
Yes	184 (20.0)	2.10 (1.01)				3.49 (0.52)			
**Q4**. **Which of the following conditions/diseases could be** cured **by an individual’s commitment to follow a healthy lifestyle?**									
− Type 1 diabetes			1.13	.288	.001		0.29	.589	.000
No	909 (98.6)	2.10 (0.88)				3.53 (0.44)			
Yes	13 (1.4)	1.86 (0.97)				3.47 (0.55)			
− Cancer			0.78	.779	.000		8.53	**.004**	.009
No	900 (97.6)	2.09 (0.87)				3.54 (0.44)			
Yes	22 (2.4)	2.12 (1.32)				3.24 (0.61)			
− Obesity			11.85	**<.001**	.013		19.50	**<.001**	.020
No	387 (42.0)	1.96 (0.78)				3.45 (0.41)			
Yes	535 (58.9)	2.18 (0.94)				3.58 (0.46)			
− HIV Infection			0.36	.850	.000		0.005	.946	.000
No	859 (93.2)	2.09 (0.87)				3.53 (0.44)			
Yes	63 (6.8)	2.15 (0.98)				3.51 (0.42)			
− Type 2 diabetes			4.07	**.044**	.004		5.86	**.016**	.006
No	541 (58.7)	2.04 (0.84)				3.50 (0.42)			
Yes	381 (41.3)	2.17 (0.93)				3.57 (0.46)			
− Osteoarthritis			1.10	.294	.003		1.73	.188	.002
No	863 (93.6)	2.09 (0.85)				3.53 (0.43)			
Yes	59 (6.4)	2.16 (1.22)				3.44 (0.60)			
**Q5. What makes it difficult for people to lose weight?**									
			7.65	**<.001**	.025		8.38	**<.001**	.027
1. Individual’s lack of motivation and or self-discipline leading to sedentary lifestyle and poor food choices	528 (57.3)	2.21 (0.99)				3.58 (0.45)			
2. Genetic and or acquired dysfunction of metabolism, not modifiable by lifestyle choices	53 (5.7)	1.67 (0.60)				3.29 (0.51)			
3. Overabundance of food and drink that promote weight gain (e.g., high sugar content)	261 (28.3)	1.99 (0.78)				3.48 (0.39)			
4. Other	80 (8.7)	1.95 (0.78)				3.46 (0.40)			
**Q6. Which of the following is the** most effective **treatment for severe obesity (BMI > 35 kg/m**^**2**^**)?**									
			0.164	.920	.001		1.88	.131	.006
1. Lifestyle interventions (diet and exercise)	258 (28.0)	2.11 (0.90)				3.51 (0.47)			
2. Medications	16 (1.7)	2.08 (1.18)				3.45 (0.40)			
3. Psychological support and behavioural modifications	257 (27.9)	2.07 (0.79)				3.52 (0.41)			
4. Surgery	391 (42.4)	2.10 (0.91)				3.55 (0.44)			
**Q7. A patient** does not lose significant weight **while participating in a** lifestyle intervention programme**. This is most likely due to...”**									
			3.87	**.009**	.013		11.92	**<.001**	.038
1. Poor compliance due to lack of motivation/self-discipline	302 (32.8)	2.20 (0.96)				3.65 (0.40)			
2. Poor compliance due to factors beyond patient’s control (i.e., working or family conditions, other health conditions for example those that limit mobility)	344 (37.3)	2.11 (0.87)				3.50 (0.47)			
3. Inadequate treatment/advise/support by care providers (i.e., physician, dietitian, exercise specialist or psychologist)	176 (19.1)	2.03 (0.80)				3.50 (0.42)			
4. Biological mechanisms of the disease (obesity) that are resistant to lifestyle intervention	91 (9.9)	1.80 (0.74)				3.32 (0.40)			
**Q8.** After losing **a significant amount of weight from a** lifestyle programme **a patient** regains **all/most of their weight. This is most likely due to...”**									
			1.68	.170	.006		3.19	**.023**	.011
1. An individual’s diet and or lifestyle choices	326 (35.4)	2.19 (0.91)				3.59 (0.43)			
2. Factors beyond patient’s control (i.e., working conditions, other Health conditions for example those that limit mobility) that undermine compliance with post-operative follow up/diet	213 (23.1)	2.09 (0.93)				3.50 (0.51)			
3. Inadequate follow up treatment/advice/support by care providers (i.e., physician, dietitian, exercise specialist or psychologist)	229 (24.8)	2.05 (0.85)				3.51 (0.39)			
4. Relapse or progression of the disease (obesity)	147 (15.9)	1.98 (0.76)				3.45 (0.41)			

Notes. Underlined words in the questions reflect emphasis present in the original ASK questionnaire and were retained as administered in the present study. Adjusted by sociodemographic variables, weight status and WBIS-M. M = mean; SD = standard deviation; N = number of participants. F = statistic from the ANOVA models. HIV = human immunodeficiency virus. Bold values indicate statistically significant results (*p* < .05). * Cohen’s d for the comparison or two means; η² effect size for more than two means

According to HCPs, the most common causes of a person overeating (Q2) are ‘Malfunction of physiological mechanisms that regulate appetite/satiety’ (36.1%; n = 333) and ‘Emotional/comfort eating’ (32.6; n = 301). There were significant differences in both the Dislike scale and the F-Scale based on the belief in the most common cause of a person overeating. Those who attributed the causes of overeating to emotional/comfort eating had higher scores than those who attributed it to malfunction of physiological mechanisms that regulate appetite/satiety, close to significance (*p* = 0.063).

When these causes were grouped into those attributable to the individual (food addiction, gluttony, and emotional/comfort eating) and other causes (food environment, malfunction of physiological mechanisms that regulate appetite/satiety, other), about 38.1% (n = 349) of HCPs attributed overeating to personal responsibility causes. These HCPs showed higher levels of weight stigma on both scales.

Although most HCPs correctly reported believing that most chronic diseases mentioned could not be entirely prevented (Q3) or cured (Q4) with an individual’s commitment to follow a healthy lifestyle, 65.6% (n = 605, prevented) and 58.9% (n = 535, cured) did report that it was possible in the case of obesity. These HCPs showed significantly higher levels of stigma on both scales compared to those who believed that obesity could not be entirely prevented or cured with lifestyle changes.

Most HCPs (57.3%; n = 528) reported believing that the main reason that makes it difficult for people to lose weight (Q5) is an individual’s lack of motivation and/or self-discipline leading to a sedentary lifestyle and poor food choices. These HCPs showed differences in their levels of weight stigma based on their beliefs. Those who attributed the difficulties in losing weight to an individual’s lack of motivation had significantly higher scores than those who attributed it to genetic and/or acquired dysfunction of metabolism, not modifiable by lifestyle choices (Dislike, *p* < .001; F-Scale, *p* < .001), overabundance of food and drink that promote weight gain (e.g., high sugar content) (Dislike, *p* = .005; F-Scale *p* = .014), or other causes (Dislike, *p* = .061). More than half of HCPs (56%, n = 515) endorsed the belief that the most effective treatment for severe obesity (Q6) is ‘lifestyle interventions’ (diet and exercise)“ or ‘psychological support and behavioral modifications’. There were no significant differences in beliefs about the most effective treatment for severe obesity and weight stigma scores.

Nearly a third (32.8%; n = 302) of HCPs believed that the main reason a person does not lose significant weight after participating in a lifestyle intervention program (Q7) is due to ‘poor compliance due to lack of motivation/self-discipline’, showing significant differences in weight stigma levels based on these beliefs. Those who attributed the difficulties in losing weight to this reason had significantly higher scores than those who thought it was due to ‘poor compliance due to factors beyond the patient’s control’ (Dislike, *p* = .001; F-Scale, *p* < .001), ‘inadequate treatment/advice/support by care providers’ (F-Scale, *p* = .002), or ‘biological mechanisms of the disease (obesity) that are resistant to lifestyle intervention’ (F-Scale, *p* < .001). Additionally, those who reported it was due to ‘biological mechanisms of the disease’ had the lowest stigma scores, which were significantly lower than ‘poor compliance due to factors beyond the patient’s control’ (Dislike, *p* = .019; F-Scale, *p* = .005) and ‘inadequate treatment/advice/support by care providers’ (F-Scale, *p* = .013).

Finally, most HCPs attributed the regain of all/most of the weight lost after a lifestyle programme (Q8) to ‘an individual’s diet and/or lifestyle choices’ (35.4%; n = 326), with these HCPs showing higher weight stigma scores than those who attributed it to ‘relapse or progression of the disease’, who had the lowest scores (F-Scale, *p* = .007).

## Discussion

This study provides the first examination of weight stigma and obesity-related beliefs among Spanish HCPs. Three main findings emerged. First, Spanish HCPs reported negative attitudes toward people living with obesity. Second, a substantial proportion endorsed beliefs that obesity is largely preventable and curable through individual lifestyle commitment, reflecting a strong attribution to personal responsibility. Third, these beliefs were significantly associated with higher levels of weight stigma.

Currently, 55.8% of Spanish adults are living with overweight or obesity [57], and therefore, whether for weight specifically or other reasons, HCPs are likely to providing care for PLWO. This finding is therefore of concern given the aforementioned evidence that weight stigma may impact the quality of and access to healthcare for PLWO [20,58]. Compared with the ASK study [24], negative attitudes appear to be higher in the Spanish HCPs of the current study (3.53 vs 3.40 F-Scale scores for HCPs). Whilst the differences were small, bariatric surgeons reported the most negative attitudes, while obesity physicians had the lowest, and older HCPs, women and those with higher weight status showed less negative attitudes. Research examining weight stigma has demonstrated differences based on demographic characteristics of respondents [59], and specifically amongst HCPs(58). In line with previous research [30], lower weight status was associated with higher stigma levels in our sample. Although some previous research have reported higher stigma among male HCPs(24,30), we did not observe statistically significant gender differences.

Despite the World Health Organization classifying obesity as a disease (WHO recognized obesity as a disease in 1948 [60]), there are longstanding debates, which have intensified in recent years. Research has highlighted that despite health authorities such as the WHO classifying obesity as a disease, many HCPs do not believe obesity is a disease [24]. For instance, a focus group study by Funk and Colleagues [61] reported that there were divergent beliefs about obesity as a disease, with the majority viewing obesity as a risk factor for other diseases. The current study finding substantiate those of Funk et al.[61] with 25% of Spanish HCPs reporting that obesity is a disease and 72% considering it a risk factor for other diseases or sometimes a disease in itself.

A large portion of them report believing that obesity and its management is primarily an issue of individual responsibility. Nearly 40% believed that the most common causes of a person overeating are attributable to the individual (emotional or comfort eating; food addiction; and gluttony), slightly higher than what the ASK study reported for the same question (36%). This belief is not aligned with empirical evidence that highlights the complex, multifactorial nature of obesity, with many factors either wholly or partially outside of an individual’s control [62,63]. Previous research has also reported that the stronger the belief that obesity is within an individual’s control, the stronger their weight stigma attitudes [59,64]. The issue of attribution is also evident in our study. With some minor differences based on the measure of weight stigma attitudes, HCPs in our sample who attributed the causes and management of obesity to personal responsibility showed significantly more negative attitudes than those who attributed it to factors beyond an individual’s control. Two third (66%) of Spanish HCPs reported a belief that obesity is entirely preventable through an individual’s commitment to follow a healthy lifestyle, and nearly 60% believe that it can be entirely cured. A similar proportion was reported in the ASK study [24] which reported that approximately 60% of HCPs from across 77 countries believe that obesity can be entirely prevented and entirely cured by merely adhering to healthy lifestyle choices. As highlighted by O’Keeffe and colleagues [24], our findings that these beliefs are associated to weight stigma, emphasizes the need for educational initiatives aimed at addressing such gaps in HCPs knowledge about the causes and treatments for obesity.

In line with these HCPs beliefs, approximately 60% reported believing that the main reason that makes it difficult for people to lose weight is an individual’s lack of motivation and/or self-discipline leading to a sedentary lifestyle and poor food choices. More than half reported believing that the most effective treatment for severe obesity is lifestyle interventions (diet and exercise) or psychological support and behavioral modifications. However, current international clinical guidelines recommend metabolic/bariatric surgery as the most effective evidence-based treatment for severe obesity, particularly for individuals with BMI ≥ 35 kg/m² with comorbidities or with BMI ≥ 40 kg/m² [65,66]. Finally, one-third of Spanish HCPs in our sample believe that the main reason a person does not lose significant weight after participating in a lifestyle intervention program is due to poor compliance due to lack of motivation/self-discipline, and a very similar proportion attributed the regain of all/most of the weight lost after a lifestyle programme to an individual’s diet and/or lifestyle choices. This finding reflects a belief that obesity is an issue of individual responsibility, and, when weight management and obesity treatment does not lead to weight loss and/or long term adherence, factors evidenced to influence health behaviors and outcomes [3,62] are overlooked, often leading to individual blame. Such attribution of blame relating to weight loss attempts, is ingrained in societal messages relating to weight management where there is a longstanding portrayal of weight loss as easily and rapid which does not correspond with empirical evidence [67,68].

This study has several strengths, adding to the empirical evidence regarding weight stigma and obesity knowledge amongst HCPs. First, this study addresses the dearth of research examining Spanish HCPs beliefs and attitudes about obesity. Akin to other regions, the prevalence of obesity in Spain is high and has been increasing. Subsequently, Spanish HCPs are spending more time providing care to PLWO both related to obesity and its associated health outcomes, and for non-obesity related reasons. Previous research has highlighted that weight stigma impacts the patient-practitioner relationship, consultations, and delivery of care [58], therefore this study is much needed to understand the experiences that PLWO in Spain may experience. Second, the study recruited a large sample of participants from a range of specialities, allowing for a comprehensive examination of beliefs and attitudes about obesity among the Spanish HCPs surveyed. Third, this study utilized more than one established weight stigma measure to assess different components of stigma, including stereotypes and prejudicial attitudes. While the AFA has been validated in Spanish samples and the F-Scale has been widely used internationally, research measuring weight stigma often relies on a single instrument. Given that weight stigma encompasses multiple components, our use of complementary measures provides a broader assessment of stigma among the Spanish HCPs surveyed. Finally, our analysis, we adjusted the results by sociodemographic variables, weight status and WBI providing insights regarding the effect of these variables on weight stigma; previous research has highlighted their relationship with weight stigma [24,30,37–39,46,47].

The study is not without its limitations. First, in addition to the validated measures of weight stigma, due to there being no instruments to measure beliefs about the causes and treatments of obesity, we employed non-validated questions. These questions were purposively developed by collaborative consensus in the original ASK study [24], where they were also acknowledged as non-validated. As in the original study, their use reflects the absence of validated instruments that adequately capture these specific belief domains. Therefore, although this approach allowed us to examine beliefs central to our research aims, the lack of formal psychometric validation may limit the interpretability and precision of these findings. Second, although the F-Scale is widely used, the adapted Spanish version employed in this study has not undergone formal psychometric validation in Spain, which should be considered when interpreting the findings. However, we used a version of the F-Scale that has been validated in Mexico, and as reported above, internal consistency in our sample was good. Third, the study relied on a voluntary convenience sample recruited through professional societies, which may introduce self-selection bias. As the survey link was disseminated by the professional and academic societies, the total number of potential respondents invited is unknown, and a response rate could not be calculated. Therefore, the findings may not be representative of all Spanish HCPs. It is possible that professionals with a particular interest in obesity or stronger views on the topic were more likely to participate, which may have influenced the overall levels of weight stigma and beliefs observed. Fourth, due to the voluntary recruitment methodology, we did not have equal demographic groups. For instance, 74% of the sample were women, and 90% provide care for PLWO. Thus, our findings may provide a greater reflection the attitudes, stigma and knowledge of HCPs who provide care for PLWO and who are women. Fifth, weight status was calculated using self-reported height and weight, which may be subject to reporting bias [69]. HCPs, like the general population, may not accurately report their weight and height, potentially leading to misclassification of BMI categories. This may have attenuated or influenced the associations observed between weight status and weight stigma. Sixth, regarding the study of HCPs’ beliefs, some subgroup categories included relatively small sample sizes; therefore, these estimates should be interpreted with caution. Seventh, our study employed explicit measures of attitudes, stigma and knowledge about obesity, and therefore responses may have been affected by social desirability and an unwillingness to provide true answers. We did not include implicit measures of weight bias (e.g., Implicit Association Test), and therefore we cannot determine whether implicit attitudes may differ from the explicit attitudes reported here. However, whilst using implicit measures of weight bias may help to address this potential limitation, there is substantial research examining this topic has employed and has demonstrated that the explicit measures used in this study show good reliability and validity. Finally, data for this study was collected in 2020. Since then, there have been continued efforts to raise awareness and address weight stigma with HCPs a key group. For instance, new guidelines and consensus statements have been published calling for action to address weight stigma in healthcare [3,7]. In Spain, new national plans for the prevention of childhood obesity have been developed [70], and important guidelines [71] have been published that promote a change in the narrative about obesity, respectful treatment of PLWO, the use of non-stigmatizing language, representations, and narratives, and the training of professionals involved in the management of obesity in stigma-free practices. These are very recent proposals, and we do not yet know the impact they may be having on HCPs in Spain.

In summary, this study provides the first insights about the attitudes, stigma and knowledge of Spanish HCPs. The findings highlight that Spanish HCPs, a high proportion of which provide care for PLWO, report weight stigma attitudes, and believe that obesity is primarily a matter of individual responsibility both with regards to the causes and treatment of obesity. Findings highlight that there is a need for HCPs education regarding the complex, multifaceted etiology of obesity, as well as the need for holistic care as part of more effective treatment that goes beyond individual responsibility. Given that it is well-evidenced that weight stigma experiences, particularly those within healthcare and from HCPs, have a detrimental impact on health (e.g., increased weight bias internalization, depression and reduced self-esteem), engagement in healthy behaviors (e.g., disordered eating behavior, reduced physical activity), as well as reduced engagement and healthcare seeking behaviors, we subsequently, call for action to address weight stigma amongst Spanish HCPs [20, 58, 72, 73]. Effective action to address weight stigma, which is pervasive across society, requires the engagement from policymakers and health systems. In line with Ewing [74], when weight stigma is experienced in healthcare, it becomes a health threat itself, which may hinder intervention efforts, and risks the exacerbation of health inequalities.

## Supporting information

S1 Checklist STROBE checklist for cross-sectional studies (PONE-D-25–40143). (DOCX)

## References

[1] World Obesity Federation. Obesity: missing the 2025 global targets. London: World Obesity Federation. 2020. http://s3-eu-west-1.amazonaws.com/wof-files/970_-_WOF_Missing_the_2025_Global_Targets_Report_ART.pdf

[2] Puhl RM, Latner JD, O’Brien K, Luedicke J, Danielsdottir S, Forhan M. A multinational examination of weight bias: predictors of anti-fat attitudes across four countries. Int J Obes (Lond). 2015;39(7):1166–73. doi: 10.1038/ijo.2015.32 25809827

[3] Rubino F, Puhl RM, Cummings DE, Eckel RH, Ryan DH, Mechanick JI, et al. Joint international consensus statement for ending stigma of obesity. Nat Med. 2020;26(4):485–97. doi: 10.1038/s41591-020-0803-x 32127716 PMC7154011

[4] Puhl RM, Andreyeva T, Brownell KD. Perceptions of weight discrimination: prevalence and comparison to race and gender discrimination in America. Int J Obes (Lond). 2008;32(6):992–1000. doi: 10.1038/ijo.2008.22 18317471

[5] Spahlholz J, Baer N, König HH, Riedel‐Heller SG, Luck‐Sikorski C. Obesity and discrimination – a systematic review and meta‐analysis of observational studies. Obes Rev. 2016;17(1):43–55. doi: 10.1111/obr.12343 26596238

[6] Pearl RL, Puhl RM, Lessard LM, Himmelstein MS, Foster GD. Prevalence and correlates of weight bias internalization in weight management: A multinational study. SSM Popul Health. 2021;13:100755. doi: 10.1016/j.ssmph.2021.100755 33718581 PMC7920853

[7] Nutter S, Eggerichs LA, Nagpal TS, Ramos Salas X, Chin Chea C, Saiful S. Changing the global obesity narrative to recognize and reduce weight stigma: A position statement from the World Obesity Federation. Obes Rev. 2023;1–9. doi: 10.1111/obr.13642 PMC13019846 37846179

[8] Brewis A, SturtzSreetharan C, Wutich A. Obesity stigma as a globalizing health challenge. Global Health. 2018;14(1):20. doi: 10.1186/s12992-018-0337-x 29439728 PMC5811962

[9] Pearl RL, Puhl RM. Weight bias internalization and health: a systematic review. Obes Rev. 2018;19(8):1141–63. doi: 10.1111/obr.12701 29788533 PMC6103811

[10] Levinson JA, Kinkel-Ram S, Myers B, Hunger JM. A systematic review of weight stigma and disordered eating cognitions and behaviors. Body Image. 2024;48:101678. doi: 10.1016/j.bodyim.2023.101678 38278088 PMC11180546

[11] Wu Y-K, Berry DC. Impact of weight stigma on physiological and psychological health outcomes for overweight and obese adults: A systematic review. J Adv Nurs. 2018;74(5):1030–42. doi: 10.1111/jan.13511 29171076

[12] Papadopoulos S, Brennan L. Correlates of weight stigma in adults with overweight and obesity: A systematic literature review. Obesity (Silver Spring). 2015;23(9):1743–60. doi: 10.1002/oby.21187 26260279

[13] Pearl RL, Wadden TA, Jakicic JM. Is weight stigma associated with physical activity? A systematic review. Obesity (Silver Spring). 2021;29(12):1994–2012. doi: 10.1002/oby.23274 34747131 PMC8612947

[14] Jackson SE, Beeken RJ, Wardle J. Obesity, perceived weight discrimination, and psychological well-being in older adults in England. Obesity (Silver Spring). 2015;23(5):1105–11. doi: 10.1002/oby.21052 25809860 PMC4414736

[15] Sutin AR, Terracciano A. Perceived weight discrimination and obesity. PLoS One. 2013;8(7):e70048. doi: 10.1371/journal.pone.0070048 23894586 PMC3722198

[16] Corrigan PW, Watson AC, Barr L. The self-stigma of mental illness: Implications for self-esteem and self-efficacy. J Soc Clin Psychol. 2006;25(8):875–84. doi: 10.1521/jscp.2006.25.8.875

[17] Telo GH, Friedrich Fontoura L, Avila GO, Gheno V, Bertuzzo Brum MA, Teixeira JB, et al. Obesity bias: How can this underestimated problem affect medical decisions in healthcare? A systematic review. Obes Rev. 2024;25(4):e13696. doi: 10.1111/obr.13696 38272850

[18] Borisova V, Stöckelová T. “This doctor knows shit about you, but the first thing he says is, ‘you need to lose some weight’”: Anti-fat bias and the contradictory effects of fat medicalization in Czech healthcare. Fat Studies. 2024;13(3):358–76. doi: 10.1080/21604851.2024.2381920

[19] Alberga AS, Edache IY, Forhan M, Russell-Mayhew S. Weight bias and health care utilization: a scoping review. Prim Health Care Res Dev. 2019;20:e116. doi: 10.1017/S1463423619000227 32800008 PMC6650789

[20] Phelan SM, Burgess DJ, Yeazel MW, Hellerstedt WL, Griffin JM, van Ryn M. Impact of weight bias and stigma on quality of care and outcomes for patients with obesity. Obes Rev. 2015;16(4):319–26. doi: 10.1111/obr.12266 25752756 PMC4381543

[21] Puhl RM, Lessard LM, Himmelstein MS, Foster GD. The roles of experienced and internalized weight stigma in healthcare experiences: Perspectives of adults engaged in weight management across six countries. PLoS One. 2021;16(6):e0251566. doi: 10.1371/journal.pone.0251566 34061867 PMC8168902

[22] Crompvoets PI, Nieboer AP, van Rossum EFC, Cramm JM. Perceived weight stigma in healthcare settings among adults living with obesity: A cross-sectional investigation of the relationship with patient characteristics and person-centred care. Health Expect. 2024;27(1):e13954. doi: 10.1111/hex.13954 39102661 PMC10790109

[23] Schorb F. Crossroad between the right to health and the right to be fat. Fat Studies. 2020;10(2):160–71. doi: 10.1080/21604851.2020.1772651

[24] O’Keeffe M, Flint SW, Watts K, Rubino F. Knowledge gaps and weight stigma shape attitudes toward obesity. Lancet Diabetes Endocrinol. 2020;8(5):363–5. doi: 10.1016/S2213-8587(20)30073-5 32142624

[25] Fang V, Gillespie C, Crowe R, Popeo D, Jay M. Associations between medical students’ beliefs about obesity and clinical counseling proficiency. BMC Obes. 2019;6:5. doi: 10.1186/s40608-018-0222-4 30766687 PMC6360739

[26] Pacanowski CR, Vizthum D, Katz SE, Skubisz C. Weight bias among undergraduate women with health-related majors: a systematic review. J Eat Disord. 2025;13(1):108. doi: 10.1186/s40337-025-01275-1 40506778 PMC12160116

[27] Täuber S, Gausel N, Flint SW. Weight Bias Internalization: The Maladaptive Effects of Moral Condemnation on Intrinsic Motivation. Front Psychol. 2018. doi: 10.3389/fpsyg.2018.01836 PMC6170635 30319517

[28] Puhl RM, Heuer CA. The stigma of obesity: a review and update. Obesity (Silver Spring). 2009;17(5):941–64. doi: 10.1038/oby.2008.636 19165161

[29] Lawrence BJ, Kerr D, Pollard CM, Theophilus M, Alexander E, Haywood D, et al. Weight bias among health care professionals: A systematic review and meta-analysis. Obesity (Silver Spring). 2021;29(11):1802–12. doi: 10.1002/oby.23266 34490738

[30] Alberga AS, Nutter S, MacInnis C, Ellard JH, Russell-Mayhew S. Examining Weight Bias among Practicing Canadian Family Physicians. Obes Facts. 2019;12(6):632–8. doi: 10.1159/000503751 31707395 PMC6940460

[31] Bøker Lund T, Brodersen J, Sandøe P. A Study of Anti-Fat Bias among Danish General Practitioners and Whether This Bias and General Practitioners’ Lifestyle Can Affect Treatment of Tension Headache in Patients with Obesity. Obes Facts. 2018;11(6):501–13. doi: 10.1159/000493373 30537717 PMC6341345

[32] Sobczak K, Leoniuk K. Attitudes of Medical Professionals Towards Discrimination of Patients with Obesity. Risk Manag Healthc Policy. 2021;14:4169–75. doi: 10.2147/RMHP.S317808 34675711 PMC8504468

[33] Tomiyama AJ, Finch LE, Belsky ACI, Buss J, Finley C, Schwartz MB, et al. Weight bias in 2001 versus 2013: contradictory attitudes among obesity researchers and health professionals. Obesity (Silver Spring). 2015;23(1):46–53. doi: 10.1002/oby.20910 25294247

[34] Kadar GE, Thompson HG. Obesity bias among preclinical and clinical chiropractic students and faculty at an integrative health care institution: A cross-sectional study. J Chiropr Educ. 2019;33(1):8–15. doi: 10.7899/JCE-17-15 30044139 PMC6417871

[35] FitzGerald C, Hurst S. Implicit bias in healthcare professionals: a systematic review. BMC Med Ethics. 2017;18(1):19. doi: 10.1186/s12910-017-0179-8 28249596 PMC5333436

[36] Liang JW, Buonpane C, Wang S, Salles A. Implicit and explicit weight bias among healthcare professionals. Int J Obes. 2025;49(11). doi: 10.1038/s41366-025-01878-3 40764653

[37] Schwartz MB, Chambliss HO, Brownell KD, Blair SN, Billington C. Weight bias among health professionals specializing in obesity. Obes Res. 2003;11(9):1033–9. doi: 10.1038/oby.2003.142 12972672

[38] Schwenke M, Luppa M, Pabst A, Welzel FD, Löbner M, Luck-Sikorski C, et al. Attitudes and treatment practice of general practitioners towards patients with obesity in primary care. BMC Fam Pract. 2020;21(1):169. doi: 10.1186/s12875-020-01239-1 32807094 PMC7433134

[39] Baska A, Świder K, Zgliczyński WS, Kłoda K, Mastalerz-Migas A, Babicki M. Is Obesity a Cause for Shame? Weight Bias and Stigma among Physicians, Dietitians, and Other Healthcare Professionals in Poland-A Cross-Sectional Study. Nutrients. 2024;16(7):999. doi: 10.3390/nu16070999 38613032 PMC11013468

[40] Caterson ID, Alfadda AA, Auerbach P, Coutinho W, Cuevas A, Dicker D, et al. Gaps to bridge: Misalignment between perception, reality and actions in obesity. Diabetes Obes Metab. 2019;21(8):1914–24. doi: 10.1111/dom.13752 31032548 PMC6767048

[41] Halford JCG, Bereket A, Bin-Abbas B, Chen W, Fernández-Aranda F, Garibay Nieto N, et al. Misalignment among adolescents living with obesity, caregivers, and healthcare professionals: ACTION Teens global survey study. Pediatr Obes. 2022;17(11):e12957. doi: 10.1111/ijpo.12957 35838551

[42] World Medical Association. World Medical Association Declaration of Helsinki: ethical principles for medical research involving human subjects. JAMA. 2013;310(20):2191–4. doi: 10.1001/jama.2013.281053 24141714

[43] World Medical Association. Obesity: preventing and managing the global epidemic. Report of a WHO consultation. World Health Organ Tech Rep Ser. 2000;894:i–xii, 1–253. 11234459

[44] Mainous AG, Yin L, Wu V, Sharma P, Jenkins BM, Saguil AA. Body mass index vs body fat percentage as a predictor of mortality in adults aged 20-49 years. Ann Fam Med. 2025;23(4):337–43. doi: 10.1370/afm.240330 40555527 PMC12306999

[45] Sheehan L, Nieweglowski K, Corrigan PW. Structures and Types of Stigma. The Stigma of Mental Illness - End of the Story?. Springer International Publishing. 2016. p. 43–66. 10.1007/978-3-319-27839-1_3

[46] Durso LE, Latner JD. Understanding self-directed stigma: development of the weight bias internalization scale. Obesity (Silver Spring). 2008;16 Suppl 2:S80-6. doi: 10.1038/oby.2008.448 18978768

[47] Pearl RL, Puhl RM. Measuring internalized weight attitudes across body weight categories: validation of the modified weight bias internalization scale. Body Image. 2014;11(1):89–92. doi: 10.1016/j.bodyim.2013.09.005 24100004

[48] Andrés A, Fornieles-Deu A, Sepúlveda AR, Beltrán-Garrayo L, Montcada-Ribera A, Bach-Faig A, et al. Spanish validation of the Modified Weight Bias Internalization Scale (WBIS-M) for adolescents. Eat Weight Disord. 2022;27(8):3245–56. doi: 10.1007/s40519-022-01453-z 35902481 PMC9333680

[49] Macho S, Andrés A, Saldaña C. Validation of the modified weight bias internalization scale in a Spanish adult population. Clin Obes. 2021;11(4):e12454. doi: 10.1111/cob.12454 33821573

[50] Anastasiadou D, Tárrega S, Fornieles-Deu A, Moncada-Ribera A, Bach-Faig A, Sánchez-Carracedo D. Experienced and internalized weight stigma among Spanish adolescents. BMC Public Health. 2024;24(1):1743. doi: 10.1186/s12889-024-19246-7 38951859 PMC11218352

[51] Crandall CS. Prejudice against fat people: ideology and self-interest. J Pers Soc Psychol. 1994;66(5):882–94. doi: 10.1037//0022-3514.66.5.882 8014833

[52] Magallares A, Morales JF. Spanish adaptation of the antifat attitudes scale/ adaptación al castellano de la escala de actitud antiobesos. Rev Psicol Soc. 2014;29(3):563–88. doi: 10.1080/02134748.2014.972707

[53] Bacon JG, Scheltema KE, Robinson BE. Fat phobia scale revisited: the short form. Int J Obes Relat Metab Disord. 2001;25(2):252–7. doi: 10.1038/sj.ijo.0801537 11410828

[54] Bacardí-Gascón M, Jimenéz-Cruz A, Castillo-Ruiz O, Bezares-Sarmiento V, León-González JM. Fobia hacia la obesidad en estudiantes Mexicanos de nutricion. Nutr Hosp. 2015;32(6):2956–7. doi: 10.3305/nh.2015.32.6.9812 26667758

[55] Crandall CS, D’Anello S, Sakalli N, Lazarus E, Wieczorkowska G, Feather NT. An attribution-value model of prejudice: Anti-fat attitudes in six nations. Pers Soc Psychol Bull. 2001;27(1):30–7. doi: 10.1177/0146167201271003

[56] Cohen J. Power analysis for the behavioral sciences. 2nd ed. Hillsdale, NJ: Erlbaum. 1988.

[57] Gutiérrez-González E, García-Solano M, Pastor-Barriuso R, Fernández de Larrea-Baz N, Rollán-Gordo A, Peñalver-Argüeso B, et al. Socio-geographical disparities of obesity and excess weight in adults in Spain: insights from the ENE-COVID study. Front Public Health. 2023;11:1195249. doi: 10.3389/fpubh.2023.1195249 37529423 PMC10387530

[58] Brown A, Flint SW, Batterham RL. Pervasiveness, impact and implications of weight stigma. EClinicalMedicine. 2022;47:101408. doi: 10.1016/j.eclinm.2022.101408 35497065 PMC9046114

[59] Flint SW, Hudson J, Lavallee D. UK adults’ implicit and explicit attitudes towards obesity: a cross-sectional study. BMC Obes. 2015;2:31. doi: 10.1186/s40608-015-0064-2 26351567 PMC4560922

[60] James WPT. WHO recognition of the global obesity epidemic. Int J Obes (Lond). 2008;32 Suppl 7:S120-6. doi: 10.1038/ijo.2008.247 19136980

[61] Funk LM, Jolles SA, Voils CI. Obesity as a disease: has the AMA resolution had an impact on how physicians view obesity?. Surg Obes Relat Dis. 2016;12(7):1431–5. doi: 10.1016/j.soard.2016.05.009 27444860 PMC6985908

[62] Butland B, Jebb SA, Kopelman P, McPherson K, Thomas S, Mardell J, et al. Tackling obesities: Future choices - project report. 2007. https://assets.publishing.service.gov.uk/government/uploads/system/uploads/attachment_data/file/287937/07-1184x-tackling-obesities-future-choices-report.pdf 10.1111/j.1467-789X.2007.00344.x 17316292

[63] Rubino F, Cummings DE, Eckel RH, Cohen RV, Wilding JPH, Brown WA, et al. Definition and diagnostic criteria of clinical obesity. Lancet Diabetes Endocrinol. 2025;13(3):221–62. doi: 10.1016/S2213-8587(24)00316-4 39824205 PMC11870235

[64] Pearl RL, Lebowitz MS. Beyond personal responsibility: effects of causal attributions for overweight and obesity on weight-related beliefs, stigma, and policy support. Psychol Health. 2014;29(10):1176–91. doi: 10.1080/08870446.2014.916807 24754230

[65] Eisenberg D, Shikora SA, Aarts E, Aminian A, Angrisani L, Cohen RV, et al. 2022 American Society for Metabolic and Bariatric Surgery (ASMBS) and International Federation for the Surgery of Obesity and Metabolic Disorders (IFSO): Indications for Metabolic and Bariatric Surgery. Surg Obes Relat Dis. 2022;18(12):1345–56. doi: 10.1016/j.soard.2022.08.013 36280539

[66] Wharton S, Lau DCW, Vallis M, Sharma AM, Biertho L, Campbell-Scherer D, et al. Obesity in adults: a clinical practice guideline. CMAJ. 2020;192(31):E875–91. doi: 10.1503/cmaj.191707 32753461 PMC7828878

[67] Flint SW. Weight stigma and discrimination: Time for change!. Nutrition Bulletin. 2019;44(3):249–53. doi: 10.1111/nbu.12398

[68] Flint SW, Vázquez-Velázquez V, Le Brocq S, Brown A. The real-life experiences of people living with overweight and obesity: A psychosocial perspective. Diabetes Obes Metab. 2025;27 Suppl 2(Suppl 2):35–47. doi: 10.1111/dom.16255 39931901 PMC12000856

[69] Fayyaz K, Bataineh MF, Ali HI, Al-Nawaiseh AM, Al-Rifai’ RH, Shahbaz HM. Validity of Measured vs. Self-Reported Weight and Height and Practical Considerations for Enhancing Reliability in Clinical and Epidemiological Studies: A Systematic Review. Nutrients. 2024;16(11):1704. doi: 10.3390/nu16111704 38892637 PMC11175070

[70] Alto Comisionado para la Pobreza Infantil. National strategic plan for the reduction of childhood obesity (2022 - 2030). Madrid: Alto Comisionado para la Pobreza Infantil. 2022. https://www.comisionadopobrezainfantil.gob.es/sites/default/files/2023-01/Plan_obesidad_Resumen_DIGITAL_ENG.pdf

[71] Lecube A, Azcona C, Azriel S, Baile JI, Barreiro E, Blay G. 2a edición guía española giro: guía española del manejo integral y multidisciplinar de la obesidad en personas adultas. Madrid: Sociedad Española para el Estudio de la Obesidad (SEEDO). 2024.

[72] Pearl RL, Sheynblyum M. How Weight Bias and Stigma Undermine Healthcare Access and Utilization. Curr Obes Rep. 2025;14(1):11. doi: 10.1007/s13679-025-00605-3 39832116

[73] Ryan L, Coyne R, Heary C, Birney S, Crotty M, Dunne R, et al. Weight stigma experienced by patients with obesity in healthcare settings: A qualitative evidence synthesis. Obesity Reviews. 2023;24(10). doi: 10.1111/obr.13606 37533183

[74] Ewing E. Weight bias and stigmatisation: what is it and what can we do about it?. Br J Gen Pract. 2019;69(684):349. doi: 10.3399/bjgp19X704405 31249087 PMC6592337

