# Correction: Stigmatizing attitudes and misconceptions about obesity among Spanish healthcare professionals

**DOI:** 10.1371/journal.pone.0361915

**Published:** 2026-10-30

**Authors:** The PLOS One Staff

Table 3 was uploaded incorrectly. Please see the correct Table 3 here.

The publisher apologizes for the error.

**Table 3:** Beliefs about causes and treatments of obesity and ANOVAs of their association with Dislike and F-Scale.

		Dislike				F-Scale			
	N (%)	M (SD)	*F*	*p*	d Cohen/ η²^*^	M (SD)	*F*	*p*	Cohen’s d/ η²^*^
**Q1. Obesity is best defined as…**									
			2.06	.084	.009		1.27	.282	.006
1. A lifestyle choice	7 (0.8)	3.00 (1.98)				3.17 (0.73)			
2. A disease	227 (24.6)	2.04 (0.85)				3.54 (0.43)			
3. A risk factor for other diseases and sometimes a disease in itself	663 (71.9)	2.10 (0.87)				3.53 (0.44)			
4. A risk factor for other diseases	14 (1.5)	2.12 (0.83)				3.58 (0.40)			
5. Other	11 (1.2)	1.99 (0.83)				3.47 (0.46)			
**Q2. What is the most common cause of a person overeating?**									
			2.34	**.040**	.013		2.91	**.013**	.016
1. Food environment	157 (17.0)	2.08 (0.74)				3.49 (0.43)			
2. Food addiction	36 (3.9)	2.38 (1.30)				3.69 (0.58)			
3. Gluttony	14 (1.5)	2.22 (0.58)				3.45 (0.66)			
4. Emotional/comfort eating	301 (32.6)	2.17 (0.96)				3.58 (0.43)			
5. Malfunction of physiological mechanisms that regulate appetite/satiety	333 (36.1)	1.98 (0.76)				3.48 (0.43)			
6. Other	81 (8.8)	2.14 (1.03)				3.54 (0.42)			
**Q2 (bis). What is the most common cause of a person overeating?**									
−			8.17	**.004**	.009		10.71	**.001**	.012
− Personal causes (Q2: 2,3,4)	349 (38.1)	2.19 (0.99)				3.59 (0.46)			
− Other causes (Q2: 1,5,6)	567 (69.1)	2.03 (0.80)				3.49 (0.53)			
**Q3. Which of the following conditions/diseases could be entirely prevented by an individual’s commitment to follow a healthy lifestyle?**									
− Type 1 diabetes			0.002	.968	.000		7.36	**.007**	.008
No	891 (96.6)	2.09 (0.88)				3.54 (0.43)			
Yes	31 (3.4)	2.08 (0.95)				3.30 (0.61)			
− Cancer			1.83	.177	.002		3.71	**.054**	.004
No	734 (79.6)	2.08 (0.86)				3.52 (0.43)			
Yes	188 (20.4)	2.15 (0.97)				3.56 (0.50)			
− Obesity			7.97	**.005**	.009		9.55	**.002**	.010
No	317 (34.4)	1.98 (0.80)				3.47 (0.41)			
Yes	605 (65.6)	2.15 (0.91)				3.56 (0.45)			
− HIV Infection			0.01	.915	.000		2.03	.151	.002
No	572 (62.0)	2.10 (0.90)				3.52 (0.45)			
Yes	350 (38.0)	2.09 (0.85)				3.55 (0.43)			
− Type 2 diabetes			2.18	.140	.002		3.96	.**048**	.004
No	354 (38.4)	2.04 (0.87)				3.50 (0.42)			
Yes	568 (61.6)	2.13 (0.89)				3.55 (0.46)			
− Osteoarthritis			0.10	.753	.000		0.73	.394	.001
No	738 (80.0)	2.09 (0.85)				3.54 (0.42)			
Yes	184 (20.0)	2.10 (1.01)				3.49 (0.52)			
**Q4**. **Which of the following conditions/diseases could be cured by an individual’s commitment to follow a healthy lifestyle?**									
− Type 1 diabetes			1.13	.288	.001		0.29	.589	.000
No	909 (98.6)	2.10 (0.88)				3.53 (0.44)			
Yes	13 (1.4)	1.86 (0.97)				3.47 (0.55)			
− Cancer			0.78	.779	.000		8.53	**.004**	.009
No	900 (97.6)	2.09 (0.87)				3.54 (0.44)			
Yes	22 (2.4)	2.12 (1.32)				3.24 (0.61)			
− Obesity			11.85	**<.001**	.013		19.50	**<.001**	.020
No	387 (42.0)	1.96 (0.78)				3.45 (0.41)			
Yes	535 (58.9)	2.18 (0.94)				3.58 (0.46)			
− HIV Infection			0.36	.850	.000		0.005	.946	.000
No	859 (93.2)	2.09 (0.87)				3.53 (0.44)			
Yes	63 (6.8)	2.15 (0.98)				3.51 (0.42)			
− Type 2 diabetes			4.07	**.044**	.004		5.86	**.016**	.006
No	541 (58.7)	2.04 (0.84)				3.50 (0.42)			
Yes	381 (41.3)	2.17 (0.93)				3.57 (0.46)			
− Osteoarthritis			1.10	.294	.003		1.73	.188	.002
No	863 (93.6)	2.09 (0.85)				3.53 (0.43)			
Yes	59 (6.4)	2.16 (1.22)				3.44 (0.60)			
**Q5. What makes it difficult for people to lose weight?**									
			7.65	**<.001**	.025		8.38	**<.001**	.027
1. Individual’s lack of motivation and or self-discipline leading to sedentary lifestyle and poor food choices	528 (57.3)	2.21 (0.99)				3.58 (0.45)			
2. Genetic and or acquired dysfunction of metabolism, not modifiable by lifestyle choices	53 (5.7)	1.67 (0.60)				3.29 (0.51)			
3. Overabundance of food and drink that promote weight gain (e.g. high sugar content)	261 (28.3)	1.99 (0.78)				3.48 (0.39)			
4. Other	80 (8.7)	1.95 (0.78)				3.46 (0.40)			
**Q6. Which of the following is the most effective treatment for severe obesity (BMI >35 kg/m** ^**2**^ **)?**									
			0.164	.920	.001		1.88	.131	.006
1. Lifestyle interventions (diet and exercise)	258 (28.0)	2.11 (0.90)				3.51 (0.47)			
2. Medications	16 (1.7)	2.08 (1.18)				3.45 (0.40)			
3. Psychological support and behavioural modifications	257 (27.9)	2.07 (0.79)				3.52 (0.41)			
4. Surgery	391 (42.4)	2.10 (0.91)				3.55 (0.44)			
**Q7. A patient does not lose significant weight while participating in a lifestyle intervention programme. This is most likely due to...”**									
			3.87	**.009**	.013		11.92	**<.001**	.038
1. Poor compliance due to lack of motivation/self-discipline	302 (32.8)	2.20 (0.96)				3.65 (0.40)			
2. Poor compliance due to factors beyond patient’s control (i.e. working or family conditions, other health conditions for example those that limit mobility)	344 (37.3)	2.11 (0.87)				3.50 (0.47)			
3. Inadequate treatment/advise/support by care providers (i.e. physician, dietitian, exercise specialist or psychologist)	176 (19.1)	2.03 (0.80)				3.50 (0.42)			
4. Biological mechanisms of the disease (obesity) that are resistant to lifestyle intervention	91 (9.9)	1.80 (0.74)				3.32 (0.40)			
**Q8. After losing a significant amount of weight from a lifestyle programme a patient regains all/most of their weight. This is most likely due to...”**									
			1.68	.170	.006		3.19	**.023**	.011
1. An individual’s diet and or lifestyle choices	326 (35.4)	2.19 (0.91)				3.59 (0.43)			
2. Factors beyond patient’s control (i.e. working conditions, other Health conditions for example those that limit mobility) that undermine compliance with post-operative follow up/diet	213 (23.1)	2.09 (0.93)				3.50 (0.51)			
3. Inadequate follow up treatment/advice/support by care providers (i.e. physician, dietitian, exercise specialist or psychologist)	229 (24.8)	2.05 (0.85)				3.51 (0.39)			
4. Relapse or progression of the disease (obesity)	147 (15.9)	1.98 (0.76)				3.45 (0.41)			

Notes. Underlined words in the questions reflect emphasis present in the original ASK questionnaire and were retained as administered in the present study. Adjusted by sociodemographic variables, weight status and WBIS-M. M = mean; SD = standard deviation; N = number of participants. F = statistic from the ANOVA models. HIV = human immunodeficiency virus. Bold values indicate statistically significant results (*p* < .05). * Cohen’s d for the comparison or two means; η² effect size for more than two mean
