# Supplementary material for: Stigmatizing attitudes and misconceptions about obesity among Spanish healthcare professionals
Source: PLoS One. 2026 Jun 18;21(6):e0351868. doi: 10.1371/journal.pone.0351868 (PMC13278479; doi:10.1371/journal.pone.0351868)
Supplement: S1 Checklist — (DOCX) [file pone.0351868.s001.docx]

# STROBE Checklist – Cross-Sectional Study

Manuscript ID: PONE-D-25-40143

Title: Stigmatizing Attitudes and Misconceptions About Obesity among Spanish Healthcare Professionals

## 1a. Study design in title/abstract

The study design (cross-sectional online survey) is clearly stated in the Abstract and in the Methods section under 'Design and Participants' (line 112).

## 1b. Informative abstract

The Abstract provides a structured and balanced summary of background, objectives, methods, key results, and conclusions (lines 22-45).

## 2. Background/rationale

The Introduction explains the scientific background of weight stigma in healthcare and the rationale for examining Spanish HCPs (lines 49-104).

## 3. Objectives

Specific objectives are clearly stated at the end of the Introduction, including assessment of stigma and beliefs and their associations (lines 104-108).

## 4. Study design

Key elements of the cross-sectional observational design are presented early in the Methods section (lines 112-114).

## 5. Setting

The setting (Spain), recruitment through professional societies, and data collection period (July–October 2020) are described in the Procedure section (lines 122-143).

## 6a. Participants

Eligibility criteria, recruitment strategy, and voluntary participation through professional scientific societies are described in the Methods (lines 114-121).

## 7. Variables

All outcomes (Dislike, F-Scale), exposures (beliefs), predictors (sociodemographic variables), and confounders (WBISM, weight status) are clearly defined in the Measures section (lines 154-235).

## 8. Data sources/measurement

Data sources and measurement methods for all variables, including validated scales and adapted instruments, are described in detail in the Measures section (p. 154-235).

## 9. Bias

Potential sources of bias (self-selection, self-reported BMI, social desirability, non-validated items) are addressed in the Limitations section (lines 455-498).

## 10. Study size

The study size reflects the largest achievable national sample during the recruitment period; as the survey link was disseminated by the participating societies and the research team did not have access to the total number of recipients, a response rate could not be calculated. This is clarified in the Methods (lines 116-118).

## 11. Quantitative variables

Quantitative variables (e.g., BMI categories, WBISM cut-offs, age groups) and their categorization are explained in the Measures (lines 154-235) and Data Analysis sections (lines 239-240 and 246-248).

## 12a. Statistical methods

ANOVAs adjusted for sociodemographic variables, weight status, and WBISM (all categorized) are described in the Data Analysis section (lines 241-248). Model assumptions, effect sizes and post hoc comparisons are described in the same section (lines 247-253).

## 12b. Subgroups/interactions

Analyses by profession, age, gender, workplace, and belief categories are described in the Data Analysis ((lines 238-239) and Results sections (tables 2 and 3).

## 12c. Missing data

Missing data handling is described in the Procedure section (forced-response design; minimal missing data) (lines 146-148)).

## 12d. Sampling strategy

The study relied on a non-probabilistic convenience sample; this is explicitly stated in the Methods (p. 114-115).

## 12e. Sensitivity analyses

No additional sensitivity analyses were conducted; the primary adjusted models are described in the Data Analysis section (lines 241-248).

## 13a. Participants flow

The total number of participants included (N=922) is reported in the Methods (line 121) and Results sections (table 3). Recruitment was not guided by a formal a priori power analysis; rather, the aim was to obtain the largest possible national sample within the recruitment period (lines 116-118).

## 13b. Non-participation

As recruitment occurred through professional societies and the total number invited was unknown, response rate could not be calculated (line 118).

## 13c. Flow diagram

## A flow diagram was not applicable because the study used a voluntary convenience sample, the total number of individuals invited was unknown, and participants self-selected into the survey (lines 114-121).

## 14a. Descriptive data

Detailed demographic, anthropometric, and professional characteristics are presented in Table 1 and described in the Results (lines 256-272).

## 14b. Missing data

The number of participants with missing weight data (n=3) is reported in the Procedure section (p. 146-148). Analysis were conducted with all available participants for each analysis.

## 15. Outcome data

Mean stigma scores (Dislike, F-Scale, WBISM) and distributions of beliefs are reported in Tables 1–3.

## 16a. Main results

Adjusted ANOVA results, including effect sizes and p-values, are reported in Tables 2 and 3.

## 16b. Category boundaries

BMI and age categories are explicitly defined in the Measures section (lines 154-235).

## 16c. Absolute risk

Not applicable, as the study did not estimate risk over time.

## 17. Other analyses

Subgroup analyses by belief categories and profession are reported in Table 3 and described in the Results (lines 302-368).

## 18. Key results

The Discussion begins with a structured summary of the three main findings aligned with study objectives (lines 371-376).

## 19. Limitations

A detailed Limitations section discusses sampling bias, self-reported BMI, non-validated items, absence of implicit measures, and dated data collection (lines 455-498).

## 20. Interpretation

The Discussion provides cautious interpretation, avoids causal language, and contextualizes findings within international literature and guidelines.

## 21. Generalisability

External validity and limited representativeness due to convenience sampling are discussed in the Limitations section (lines 466-476).

## 22. Funding

Funding sources and the role of funders are disclosed in the Funding section (lines 526-531).
